# An Association between Bevacizumab and Recurrent Posterior Reversible Encephalopathy Syndrome in a Patient Presenting with Deep Vein Thrombosis: A Case Report and Review of the Literature

**DOI:** 10.1155/2012/819546

**Published:** 2012-11-29

**Authors:** Mark Lazarus, Stanley Amundson, Rajesh Belani

**Affiliations:** ^1^Office of Medical Education, 4077 5th Avenue, San Diego, CA 92103, USA; ^2^3075 Health Center Drive, Suite 102, San Diego, CA 92123, USA

## Abstract

*Background*. The posterior reversible encephalopathy syndrome (PRES) is a syndrome characterized by hypertension, headache, seizures, and visual disturbances. Causes of PRES include preeclampsia/eclampsia, hypertension, and recently bevacizumab, a monoclonal antibody vascular endothelial growth factor (VEGF) inhibitor. There is no information to date about PRES recurrence in patients taking bevacizumab or descriptions of deep vein thrombosis (DVT) in the setting of PRES. We reviewed data on a patient receiving bevacizumab who presented with a DVT and PRES and later developed recurrent PRES. *Case*. A 72-year-old man with metastatic pulmonary adenocarcinoma received maintenance bevacizumab following six cycles of carboplatin and paclitaxel. Following his eighth dose of bevacizumab, he developed a DVT as well as PRES. He made a rapid recovery and was discharged from the hospital but went on to develop PRES recurrence nine days following his original episode. *Conclusion*. Several mechanisms exist whereby exposure to bevacizumab could be related to the development of both DVT and PRES by inducing global endothelial dysfunction. Recurrent PRES may result from bevacizumab's prolonged half-life (11–50 days) and suboptimal blood pressure control. In the setting of bevacizumab, PRES surveillance may play a similar role in preeclampsia screening as both diseases share similar antiangiogenic signaling pathways.

## 1. Introduction

The posterior reversible encephalopathy syndrome (PRES), initially described by Hinchey and colleagues, is a syndrome clinically characterized by hypertension, headache, confusion, visual disturbances, and seizures. Imaging findings include subcortical edema, predominantly involving the parietal and occipital lobes [1]. The mechanism is not entirely understood but is likely related to hyperperfusion with blood-brain barrier breakthrough, extravasation of fluid containing blood or macromolecules, and resulting cortical and subcortical edema [2]. Causes of PRES include eclampsia, hypertension, immunosuppressive agents, and cytotoxic chemotherapy. Most recently, bevacizumab, a monoclonal antibody that binds to the vascular endothelial growth factor (VEGF) has been linked to PRES [1, 3]. 

We describe a patient with PRES who presented with a lower-extremity deep vein thrombosis (DVT) on the day of bevacizumab infusion and developed recurrent and fatal PRES. PRES recurrence has been described in various patient populations. However, there is no information about recurrence of this syndrome in patients receiving bevacizumab [4]. 

## 2. Case Report

**Figure 1:**
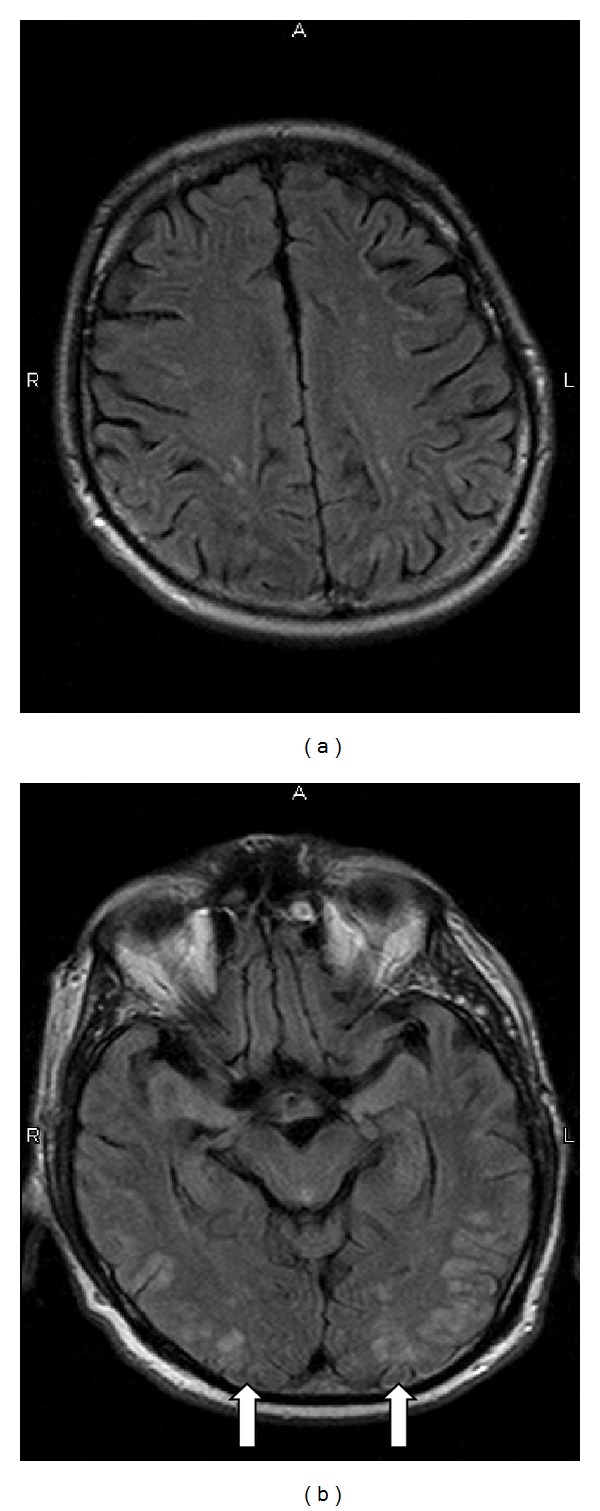
FLAIR AXIAL MRI images (TR 4140, TE 117) of the initial admission demonstrating multiple small bilateral cortical hyperintensities especially involving the occipital lobes (arrows). There was no evidence of mass effect or contrast enhancement on postcontrast images (not shown).

A 72-year-old man with metastatic pulmonary adenocarcinoma received six cycles of carboplatin, paclitaxel, and bevacizumab achieving stable disease. Subsequently the patient was placed on bevacizumab maintenance. Therapy was well tolerated with minimal toxicity. Most notably, the patient did not develop bevacizumab-induced hypertension. On the day of his eighth bevacizumab dose, he developed new-onset swelling of the right lower extremity. A lower-extremity Doppler ultrasound showed a deep vein thrombosis, and the patient was sent to the emergency department for further management. In the ED, the patient developed emesis, aphasia, altered mental status, and severe agitation. The physical exam was notable for a blood pressure (BP) of 164/75, myoclonus, tonic-clonic seizures, and bilateral upgoing Babinski responses. Laboratory assessment with urinalysis revealed a protein level of 100 mg/dL. Magnetic resonance imaging (MRI) of the brain demonstrated multiple small bilateral cortical hyperintensities, especially involving the occipital lobes and cerebellar hemispheres consistent with leukoencephalopathy (the patient had a normal MRI of the brain as a part of the staging workup prior to chemotherapy initiation) (Figure 1). Lumbar puncture was performed, with cerebrospinal fluid that was clear, colorless, and mostly acellular. CSF's total protein level was 76 mg/dL and glucose level of 76 mg/dL. The patient was admitted to the hospital after developing status epilepticus. He was intubated and received propofol for sedation. The patient was extubated seventy-two hours later and subsequently made a rapid recovery. He was discharged on hospital day 7 and was alert, at his baseline mental status, his BP measuring 134/69, and the Babinski extensor responses reverted to flexor. 

**Figure 2:**
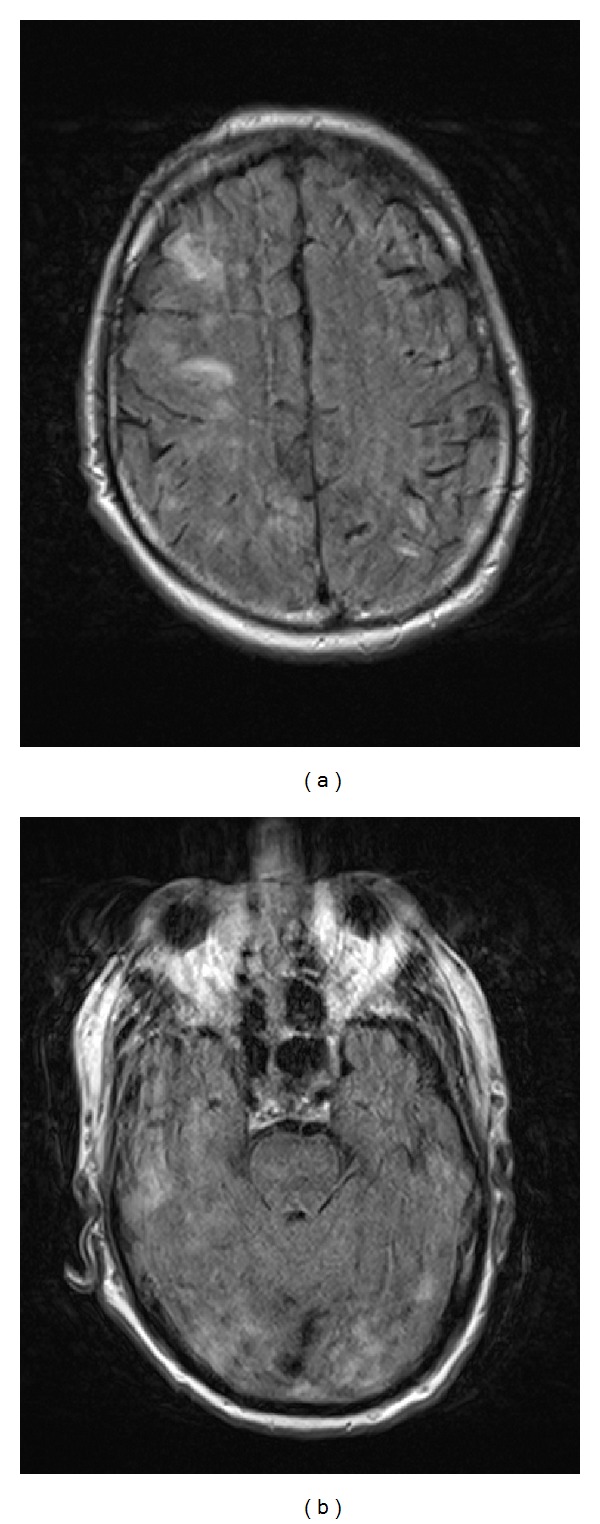
FLAIR AXIAL MRI images (TR 9000, TE 112) from the second admission demonstrating significant interval worsening of bilateral cortical hyperintensities throughout both cerebral hemispheres. Note the mild patient motion artifact.

Four days following discharge, the patient was re-admitted after suffering tonic-clonic seizure at home, accompanied by severe agitation. At presentation he was unresponsive, had an elevated BP of 161/89, and developed status epilepticus. He received propofol and was subsequently intubated. During his hospitalization, a repeat MRI showed significant interval worsening of bilateral cortical hyperintensities, ranging up to two centimeters, throughout both cerebral hemispheres (Figure 2). The cerebrospinal fluid from lumbar puncture had a protein level of 88 mg/dL and a glucose level of 61 mg/dL. Urinalysis revealed a protein level of 100 mg/dL. He developed recurrent DVT despite treatment with a therapeutic dose of enoxaparin. The patient never regained his prehospitalization mental status. Eleven days after admission, a decision was made to place the patient on comfort care. He was extubated and subsequently died. 

## 3. Discussion

PRES is a clinicoradiological syndrome characterized by headache, confusion, visual disturbance, and seizures accompanied by subcortical edema, predominantly involving the parietal and occipital lobes [1]. The term describes a reversible imaging appearance and a diverse array of presenting symptoms related to a hyperperfusion state with blood-brain barrier breakthrough, extravasation of fluid containing blood or macromolecules, and resulting cortical and subcortical edema [4].

Bevacizumab is a monoclonal antibody directed against VEGF that decreases tumor perfusion, vascular volume, and microvascular density and improves survival in patients with colorectal carcinoma and nonsquamous non-small-cell lung carcinoma [5]. However, bevacizumab-based chemotherapy is associated with a risk of grade 3 hypertension in up to sixteen percent of patients [6]. Severe hypertensive encephalopathy can lead to PRES and vasogenic edema of the posterior cerebral white matter, induced by endothelial dysfunction and a disrupted blood-brain barrier [6]. We speculate that bevacizumab induced hypertension and vasogenic edema in a patient with a dysfunctional cerebral vasculature accommodation caused by VEGF inhibition, thereby resulting in PRES.

In the clinical setting, the risk of developing DVT in patients receiving bevacizumab for the treatment of advanced solid tumors has been well reported [7]. The development of DVT may result from the anti-VEGF effect of bevacizumab whereby VEGF inhibition enhances coagulation by exposing subendothelial procoagulant phospholipids, reduces production of nitric oxide and prostacyclin by endothelial cells, increases hematocrit and blood viscosity via overproduction of erythropoietin, increases expression of proinflammatory cytokines, and possibly increases the release of procoagulant from the tumor into the blood stream due to an enhanced cytotoxic effect [8–11]. Alternatively, the increased risk of DVT may be secondary to prolonged survival with bevacizumab, although this has not been proven in non-small-cell lung cancer [12].

We describe a patient who presented with new onset hypertension, proteinuria, hyperreflexia, seizures, PRES, and a DVT shortly after the infusion ofhis eighth course of bevacizumab. We suggest that the near-simultaneous development of both DVT and PRES in our patient was due to the anti-VEGF effect of bevacizumab. Several mechanisms may be contributory. First, we speculate that bevacizumab's VEGF inhibition and decreased trophic effect on capillary endothelium caused systemic capillary damage leading to PRES. VEGF inhibition—by the aforementioned mechanisms—caused a global endothelial cell disruption leading to the development of a prothrombotic state and DVT. In addition, bevacizumab likely contributed to the acute hypertensive episode in a patient with otherwise well-controlled BP, a known adverse effect of this medication. Finally, we speculate that bevacizumab's disruption of the cerebral endothelium leading to an inappropriate vasculature accommodation to the relatively mild hypertensive episode, which in addition to the hypercoaguable state, precipitated the near-simultaneous induction of both PRES and DVT. 

PRES recurrence in our patient likely resulted from bevacizumab's ongoing anti-VEGF effect (half-life 11–50 days) in the setting of suboptimal BP management. At the time of his original discharge, the patient's BP (134/69) was still elevated compared to his former baseline (110/80). The inability to reduce the patient's BP to previous baseline levels may have resulted in continued vasogenic edema and protein extravasation through an already dysfunctional blood-brain barrier owing to bevacizumab's ongoing trophic effects. Thus, intensive BP control to pre-bevacizumab levels in patients with bevacizumab-induced PRES may help prevent PRES recurrence until VEGF levels have normalized.

This presentation is similar to preeclampsia in both clinical features and pathophysiology [13]. In the preeclampsia setting, low serum VEGF levels combined with elevated levels of VEGF inhibitors, such as soluble FLT-1 and soluble endoglin (sEng), cause cerebral endothelial damage and symptoms of PRES [14–16]. Serological studies in preeclampsia have implicated elevated serum levels of sVCAM-1, a soluble adhesion molecule associated with leukocyte trafficking, in the pathophysiology of preeclampsia [17–19]. In cancer patients who were treated with VEGF inhibitor, plasma level of sVCAM-1 was reported to be elevated as well [20]. Since both preeclamptic patients and those receiving VEGF inhibitors are in the condition of disturbed physiological angiogenesis, these soluble factors are likely to reflect endothelial dysfunction in both processes [21]. 

While monitoring for hypertension and proteinuria is a hallmark of preeclampsia screening, there are no guidelines for monitoring patients taking bevacizumab or other agents widely known to induce PRES. To date, alterations in absolute levels of VEGF, sFlt-1, and sEng offer, a promising approach for predicting preeclampsia and thus might be useful in PRES screening; however, these tests are not routinely used in the clinical setting [16, 22]. The relationship warrants further investigation to determine if screening for hypertension, proteinuria, and hyperreflexia in the setting of bevacizumab could be useful inanalogous fashionto the current standard practice ofmonitoring pregnant patients as these changes precede both syndromes. Withholding bevacizumab to patients exhibiting early manifestations of PRES could plausibly reduce the likelihood of PRES occurrence or recurrence. 

In conclusion we have identified a patient in our clinical practice with a temporal relationship between bevacizumab exposure, DVT, and recurrent and fatal PRES. Although this is a preliminary observation, these data should serve as a warning to clinicians who encounter PRES in the setting of bevacizumab as the syndrome may recur and become fatal, particularly in the setting of suboptimal BP control. In addition, PRES surveillance in cancer patients treated with VEGF inhibitors may play a similar role to preeclampsia screening in pregnant patients as both diseases may share similar antiangiogenic signaling pathways. While investigational studies of the VEGF-related factor levels remain promising, routine screening for clinical warning signs of PRES (hypertension, proteinuria, and hyperreflexia) may offer a practical surveillance strategy whereby discontinuing the offending agent and aggressively controlling BP may prevent PRES or PRES recurrence.
